# Correlation Between the Insall-Salvati Ratio and Functional Outcomes After Total Knee Arthroplasty

**DOI:** 10.7759/cureus.103416

**Published:** 2026-02-11

**Authors:** Amandeep S Bakshi, Davinder Singh, Jaspreet Singh, Sudhanshu Arya, Mukul Sharma

**Affiliations:** 1 Orthopaedics, Rajindra Hospital, Government Medical College, Patiala, Patiala, IND

**Keywords:** insall salvati ratio, insall-salvati ratio, primary total knee replacement, total knee arthroplasty, total knee arthroplasty (tka), total knee replacement (tkr)

## Abstract

Background

Total knee arthroplasty (TKA) is a highly successful and cost-effective orthopedic procedure. This study aimed to assess the correlation between the Insall-Salvati ratio (ISR), a reliable measure of patellar height, and TKA outcomes.

Methodology

This prospective observational study was conducted at a tertiary care hospital in India for one year. Consecutive patients undergoing primary TKA for degenerative knee conditions were included. A total of 200 knees were analyzed. Patellar height was assessed using the ISR on standardized lateral knee radiographs obtained at approximately 30° of flexion, both preoperatively and postoperatively. Clinical outcomes were assessed using range of motion, pain scores, Knee Society Scores (KSS), and stair-climbing ability. Statistical analysis was performed to evaluate the correlation between ISR changes and postoperative functional outcomes.

Results

The study evaluated 200 TKAs in 122 males and 78 females with a mean age of 67.8 years. Postoperatively, 17.5% of knees showed patella baja, 79% had normal ISR, and 3.5% exhibited patella alta. Among knees initially classified as normal or patella alta, 10.67% developed postoperative patella baja. Females were twice as likely as males to develop new-onset patella infera, with a higher incidence (12.8% vs. 8.19%). No significant difference in ISR reduction or clinical outcomes was observed between posterior cruciate substituting and retaining implants. However, reduced ISR correlated with poorer stair-climbing and functional scores, while KSS, pain, and flexion remained unaffected. Patients whose preoperative patella baja normalized postoperatively showed functional improvement in stair-climbing.

Conclusions

TKA led to a reduction in patellar tendon length, reflected by a decreased ISR, in approximately half of the patients. This reduction was associated with significantly lower postoperative stair-climbing and function scores, while KSS, pain levels, and range of motion remained unaffected. Patella infera was twice as prevalent in female patients compared to males.

## Introduction

Total knee arthroplasty (TKA) is widely regarded as one of the most successful, cost-effective, and transformative procedures in orthopedic surgery. It has demonstrated remarkable consistency in delivering favorable clinical outcomes, particularly in patients suffering from advanced knee joint pathology. Patient-reported outcome measures consistently highlight TKA’s substantial benefits in alleviating chronic pain, restoring joint function, and enhancing overall quality of life [1,2]. The procedure is especially effective for individuals with end-stage osteoarthritis, including tricompartmental or degenerative forms, where conservative management options have failed. By replacing the damaged articular surfaces with prosthetic components, TKA significantly improves mobility, reduces disability, and enables patients to return to daily activities with greater independence and comfort [3,4]. Among patients scheduled to undergo TKA, it is essential to obtain all anthropometric variables. To evaluate patellar height, a range of measurement techniques and imaging modalities are commonly employed in orthopedic and radiologic practice. Among these, the Insall-Salvati ratio (ISR) is one of the most frequently used and well-established methods due to its simplicity, reproducibility, and clinical relevance [5]. This technique involves comparing the length of the patellar tendon to the length of the patella on a lateral radiograph of the knee, typically taken in 30° of flexion [6,7]. The primary objective of this study was to evaluate the association between change in the ISR and postoperative functional outcomes following primary TKA. Secondary objectives included determining the incidence of postoperative patella infera, assessing sex-based differences in ISR changes, and evaluating whether implant design influenced ISR change or functional outcomes. Functional outcomes assessed included stair-climbing ability, functional score, pain score, range of motion, and Knee Society Scores (KSS).

## Materials and methods

Study design and setting

This was a prospective observational study conducted at a tertiary care hospital in India, evaluating patients who underwent TKAs for one year.

Study population

The study included 200 TKAs, including 122 men and 78 women. The average age of the patients was 67.8 years. A sample size of 200 knees was considered adequate to detect clinically meaningful correlations between changes in ISR and postoperative functional outcomes, based on prior published studies evaluating patellar height after TKA. The average body mass index was 30.2 kg/m².

Inclusion and exclusion criteria

Patients were enrolled in the study if they underwent primary TKA for degenerative knee conditions, including osteoarthritis, rheumatoid arthritis, or osteonecrosis, during the study period. Eligible patients underwent follow-up for a duration of six months. Patients were excluded if they underwent revision TKA, had a history of previous knee surgery affecting the extensor mechanism or patellar tendon length, or developed periprosthetic infection, periprosthetic fracture, or other major postoperative complications that could influence patellar height measurements or functional outcome assessment.

Radiographic evaluation

Standardized radiographs were obtained pre- and postoperatively to assess the ISR. Radiographic analysis involved measuring the patellar tendon length and patellar height on lateral radiographs obtained at approximately 30° of flexion; however, formal inter- and intra-observer reliability analysis was not performed. ISR values were categorized as patella baja (ISR < 0.8), patella alta (ISR > 1.5), or normal.

Clinical outcome assessment

Clinical outcomes, including range of motion, pain, KSS [8], and stair-climbing ability, were recorded during follow-up visits and stored in a patient database. Postoperative radiographic and functional assessments were performed at a minimum follow-up of six months for all patients. Stair-climbing ability and pain assessment were recorded as part of routine postoperative clinical evaluation using standardized institutional assessment protocols.

Statistical analysis

Statistical analysis was performed using SPSS Statistics for Windows, version 26.0 (IBM Corp., Armonk, NY, USA). Continuous variables were expressed as means and standard deviations, while categorical variables were presented as frequencies and percentages. Linear regression analysis was used to evaluate the association between changes in ISR and postoperative functional outcomes. Log-linear regression was applied for stair-climbing and pain scores. A p-value <0.05 was considered statistically significant. Power analysis confirmed that the study was adequately powered to detect differences explaining at least 1% of variance in postoperative functional outcomes, ensuring statistical reliability. Knees were considered the unit of analysis. In patients who underwent bilateral procedures, both knees were included in the analysis. The potential for statistical clustering was acknowledged as a limitation of the study.

Ethics considerations

This study was approved by the Government Medical College, Patiala Institutional Ethics Committee (approval number: Trg.9(310)2025/1424; dated 9/1/2025).

## Results

A total of 200 TKAs were analyzed. Postoperatively, 35 (17.5%) knees demonstrated patella infera, 158 (79%) knees had normal ISR, and seven (3.5%) knees exhibited patella alta. The distribution of preoperative and postoperative ISR categories is shown in Table 1.

**Table 1:** Preoperative and postoperative ISR classification. ISR: Insall-Salvati ratio

Preoperative	Postoperative			Total
	Patella infera	Normal ISR	Patella alta	
Patella infera	16 (72.73%)	6 (27.27%)	0 (0%)	22 (100%)
Normal ISR	18 (10.47%)	151 (87.89%)	3 (1.74%)	172 (100%)
Patella alta	1 (16.67%)	1 (16.67%)	4 (66.67%)	6 (100%)
Total	35 (17.5%)	158 (79%)	7 (3.5%)	200 (100%)

Among knees with normal or elevated preoperative ISR, 10.67% developed postoperative patella infera. Females demonstrated a significantly higher incidence of postoperative patella infera compared to males (12.8% vs. 8.19%). No statistically significant difference in ISR reduction was observed between posterior-stabilized and cruciate-retaining implants.

Correlation analysis

Linear regression analysis demonstrated a significant association between a reduction in ISR and postoperative stair-climbing scores (β = −0.24, p = 0.002) and functional scores (β = −0.21, p = 0.005). No significant correlation was observed between ISR reduction and KSS, pain scores, or postoperative range of motion (p > 0.05 for all). These findings are summarized in Table 2.

**Table 2:** Association between change in Insall-Salvati ratio and postoperative outcomes. P-values <0.05 are considered statistically significant.

Outcome measure	Regression coefficient	Statistical test	P-value
Stair-climbing score	-0.24	Linear regression	0.002
Functional score	-0.21	Linear regression	0.005
Knee Society Score	-0.08	Linear regression	0.41
Postoperative knee flexion	-0.05	Linear regression	0.53
Pain score	-0.06	Log-linear regression	0.47

## Discussion

TKA is widely recognized as the standard treatment for end-stage knee osteoarthritis. It is also indicated in several other conditions, such as inflammatory arthritis, post-traumatic arthritis, or deformity following fractures, congenital dysplasia, and certain malignancies. Since its inception in the early 1970s at the Hospital for Special Surgery, TKA has undergone significant advancements in design, technique, and implant materials over the past five decades. These innovations have greatly improved surgical outcomes and patient satisfaction. With the rising global burden of knee arthritis, the demand for TKA continues to escalate. Current projections estimate that the number of primary TKA procedures will increase by approximately 85%, reaching 1.26 million cases annually by the year 2030, reflecting its growing importance in orthopedic care [9-11]. Hence, the present study was conducted to assess the correlation between ISR and TKA.

This study demonstrated that TKA is associated with a reduction in patellar tendon length, as reflected by a decrease in ISR in approximately half of the patients. Postoperative patella infera was observed in 17.5% of knees and was twice as prevalent among female patients. A reduction in ISR was significantly associated with inferior stair-climbing ability and functional outcomes, while knee scores, pain levels, and range of motion were unaffected. These findings align with those reported by Meneghini et al., who similarly observed that ISR reduction adversely affected functional performance without influencing pain or knee scores [12].

The lack of difference between posterior-stabilized and cruciate-retaining implants suggests that implant design alone does not significantly influence patellar tendon shortening. These results align with the study reported by Ishii et al., which investigated longitudinal changes in the length of the patellar tendon using the ISR following bilateral TKA with different mobile-bearing designs, i.e., meniscal-bearing and rotating-platform, implanted in contralateral knees. In a cohort of 51 patients undergoing staged bilateral TKA, ISR was assessed preoperatively and at multiple postoperative intervals up to ≥5 years. The study found no significant difference in ISR change patterns between implant types; however, ISR significantly declined in both groups by six months. Meniscal-bearing knees consistently showed lower ISR values compared to rotating-platform knees throughout follow-up. ISR stabilized after the first postoperative year, and no significant correlation was observed between ISR values and clinical outcomes, suggesting that while length of the patellar tendon shortening occurs post-TKA, it may not directly affect long-term function [13]. In a study by Chen et al., 101 mobile-bearing TKA revisions were analyzed to compare the incidence of patella baja in aseptic (n = 67) versus septic (n = 34) revision cases. The preoperative ISR values were comparable between groups at 1.00 for aseptic and 0.96 for septic. The postoperative ISR was significantly lower in the septic group (0.77 vs. 0.99). After adjusting for baseline patellar height, the corrected ISR remained significantly reduced in septic cases (0.82) compared to aseptic ones (1.09). Septic revisions also showed significantly lower mean postoperative range of motion (92.2° vs. 108.0°). Additionally, patients with postoperative patella baja demonstrated reduced range of motion (95.1°) compared to those without (106.8°), indicating that septic TKA revisions using non-articulating spacers are associated with a greater risk of patella baja and impaired postoperative mobility [14].

Limitations

This study has several limitations. First, it was conducted at a single tertiary care center, which may limit the generalizability of the findings to other populations and surgical settings. Second, the minimum follow-up duration was limited to six months, which may be insufficient to assess long-term patellar tendon remodeling and functional adaptation following TKA. Third, details related to surgical technique, patellar resurfacing strategy, component positioning, and postoperative rehabilitation protocols were not independently analyzed, and these factors may influence patellar height and functional outcomes. Fourth, inter- and intra-observer reliability for ISR measurements was not formally assessed. Additionally, both knees from patients undergoing bilateral procedures were included as independent observations, which may introduce clustering effects and potentially influence statistical significance. Finally, potential confounding variables such as body mass index, quadriceps strength, and preoperative deformity were not specifically evaluated. These limitations should be considered when interpreting the results. Future prospective multicenter studies with longer follow-up are required to further clarify the impact of patellar height changes on functional outcomes after TKA.

## Conclusions

In this prospective observational study, TKA was associated with a reduction in patellar tendon length, as reflected by a decrease in ISR, in a substantial proportion of patients. A reduction in ISR demonstrated a significant association with poorer postoperative stair-climbing ability and functional scores, while pain levels, range of motion, and KSS were not significantly affected. Postoperative patella infera was observed more frequently in female patients. These findings suggest that changes in patellar height following TKA may influence activity-specific functional outcomes. However, given the observational design, short follow-up duration, and presence of unmeasured confounders, causal relationships cannot be inferred. Further multicenter studies with longer follow-up and standardized assessment protocols are required to better define the clinical significance of ISR changes after TKA.
